# Predictive Value of Technical Throwing Skills on Nomination Status in Youth and Long-Term Career Attainment in Handball

**DOI:** 10.1186/s40798-021-00397-5

**Published:** 2022-01-14

**Authors:** Till Koopmann, Franziska Lath, Dirk Büsch, Jörg Schorer

**Affiliations:** Institute of Sport Science, Carl von Ossietzky University Oldenburg, Ammerländer Heerstraße 114-118, 26129 Oldenburg, Germany

**Keywords:** Talent, Technique, Skill development, Athletic performance, Expertise, Coach’s eye

## Abstract

**Background:**

Research on talent in sports aims to identify predictors of future performance. This study retrospectively investigated 1) relationships between young handball field players’ technical throwing skills and (a) their potential nomination to youth national teams and (b) their long-term career attainment 10 years later, and 2) associations between nomination status and career attainment.

**Results:**

Results from retrospectively predicting nomination status and career attainment using logistic regression analyses show that technical throwing skills were partly able to explain players’ nomination status (Nagelkerke *R*^*2*^: females 9.2%, males 13.1%) and career attainment (Nagelkerke *R*^*2*^: 9.8% for female players). Here, variables throwing velocity and time on exercise showed statistically significant effects. In addition, nomination status and career attainment were shown to be associated using chi-square tests (*w* of .37 and .23 for female and male players, respectively) and nomination status as a predictor increased the prediction of career attainment remarkably (Nagelkerke *R*^*2*^: females 20.3%, males 12.7%).

**Conclusions:**

Given these results, basic technical throwing skills may serve rather as a prerequisite in this age group on national level, emphasizing its importance already on lower levels and in younger age groups. Furthermore, advantages from entering the national TID system early especially for females are discussed.

## Key Points

- Technical skills can help to predict nomination status and career attainment.
- Throwing velocity can help to predict nomination status and career attainment.
- Nomination to youth national teams is a strong predictor of career attainment.
- Female players are more dependent on the federation’s talent development system

## Introduction

Most coaches, scouts and researchers agree that performance as well as talent in sport is multidimensional concepts depending on a variety of aspects (e.g., technical, tactical, social, cognitive/psychological, physical/physiological) [1, 2]. In recent years, research in the field of talent has investigated many potential aspects and factors in various areas mostly conducting cross-sectional studies. While this gives valuable insights and expands the understanding of underlying processes and mechanisms, the field lacks longitudinal data and studies allowing for a better understanding as the concept of talent is by nature longitudinal.

The assessment of young athletes and their potential for superior future sport performance is widely conducted following multidimensional approaches [1, 3, 4]. Talent identification and development (TID) programs in many sports aim to identify ‘talented’ young athletes based on two approaches: 1) coaches’ decisions including observations (i.e., ‘coach’s eye’) and/or 2) results from mostly physical and (sport specific) technical tests (i.e., testing combines or batteries). On the one hand, despite being an integral part of TID, the concept of ‘coach’s eye’ is largely unknown and rather vague to date as only few studies investigated the underlying experiences, processes and contexts of coaches’ decision-making [5–7]. On the other hand, the use of testing combines in both practical and scientific contexts to identify or discriminate talented young athletes is widespread. In many cases, testing combines focus on the assessment of anthropometric (e.g., standing and sitting height, hand size) and/or physical and physiological parameters (e.g., endurance, speed, agility, strength) that can be strongly affected by age and maturation processes [8]. These differences in anthropometric and physical parameters can lead to situations in that older [‘relative age effects’; 9] and early maturing players [8] have higher chances to be selected. Here, it is important to note that differences in maturation and ‘relative age effects’ are closely related but different concepts that should be separated [10]. These factors emphasize the individual and dynamic nature of both performance and talent that always needs to be considered when assessing young athletes. Previous research combined and compared both approaches in handball finding the best model-fit regression based on motor-tests outperformed retrospective predictions by national and regional coaches as well as novice and advanced handball players [11]. However, although only with a small difference, national coaches showed the best retrospective predictions based on ‘coach’s eye.’ That is, (national) coaches were at least to some degree able to predict future performance based on their observations, suggesting a positive association between nomination in youth and later career attainment, but the combination of various objective test measures appeared more promising. Here, also the combination of both objective and subjective measures should be considered and investigated further as it showed the best prediction of player status 5 years after assessment compared to objective and subjective measures separately in elite youth football [12]. In another study investigating coaches’ decision-making in talent contexts, Koz, Fraser‐Thomas and Baker [13] found between 3 and 17% of variance in later success to be explained by draft round in the four major sports in North America. Furthermore, Cripps, Hopper and Joyce [14] had nine Australian football coaches prospectively predict their 264 adolescent athletes’ level of career attainment (semi-elite adolescent, semi-elite senior, professional senior) 4 years later. They found a fair level of agreement with the coaches predicting 63% of the athletes correctly. This indicates coaches’ fair capacity to predict players’ development when knowing the players well for a long time. Finally, Cripps, Banyard, Woods, Joyce and Hopper [15] assessed eight physical and anthropometrical variables in 80 adolescent males under 16 Australian footballers and used these to test for differences between professional and non-professional athletes at under 18. The authors found statistically significant differences for the 20-m sprint outcomes, while a binary logistic regression was unable to predict an effect of any measure on career attainment [15].

In addition to anthropometric and physical/physiological measurements, TID programs try to incorporate assessments of sport-specific technical skills in their test batteries. In their recent systematic review, Koopmann, Faber, Baker and Schorer [16] found almost all (93%) of the 59 studies analyzed in their study reported discriminatory, explanatory and/or predictive benefits for the assessment of technical skills in talented youth athletes. For all studies in the systematic review, assessments of technical skills were categorized based on their method type (‘technique-related’ or ‘outcome-related,’ describing an assessed variable’s focus) and their method setup (‘experimental’ or ‘competition,’ describing the data acquisition’s environment or context, this way assessing representativeness) [16]. Despite identifying areas of limited knowledge in regard to different populations (e.g., female sports and sports other than soccer) and a predominance of specific assessment methods (i.e., ‘outcome-related’ method type and ‘experimental’ method setup), the authors concluded assessing technical skills in the context of TID generally appears valuable and promising in many sports.

Only few studies investigated technical skills of talents in handball. As part of the nomination to the junior Israeli national team, Lidor, Falk, Arnon, Cohen, Segal and Lander [17] conducted a test battery consisting of anthropometrical (i.e., height, weight), physical (i.e., running, explosive power, speed) and technical skills (i.e., slalom dribbling test) measurements with 405 12–16 years old female and male handball field players. Comparing the nominated and non-nominated players, the only measurement shown to be a good indicator of nomination was technical dribbling skill measured following an ‘outcome-related’ method type and an ‘experimental’ method setup as the variable was the time needed to finish a 15 m-slalom course with five cones. Similarly, Matthys et al. [18] found statistically significant differences over a 3-year period for, among other variables, slalom dribbling skills in 94 Belgian non-elite and elite youth handball players (13–17 years of age). In another study, Naisidou, Kepesidou, Kontostergiou and Zapartidis [19] compared the testing combine results of successful and less successful Greek elite players (13 ± 0.5 years of age). Analyzing anthropometrical, physical and technical characteristics applying ‘outcome-related’ method type and an ‘experimental’ method setup, they found successful players had a higher body height, a greater hand span and showed significantly better performance in, among others, ball throwing velocity, slalom dribbling and defensive movement. In a third study, Schorer et al. [20] assessed both technical and tactical skills in female handball players (14.4 ± 0.4 years of age) using notational analyses during (small-sided) games (‘outcome-related’ method type, ‘competition’ method setup) at a talent selection camp and related these to the players’ highest league as a proxy for career attainment 18 years after the camp. Results showed significant differences between the three groups (professional, semi-professional and non-professional adult players) only for the number of actions taken, not the quality of those actions. This might be explained by the most talented players taking more actions also in difficult situations whereas less talented players take less actions in clearer and easier situations.

In summary, these three studies emphasize the important role of technical skills in TID in handball, but also the limited knowledge regarding both their predictive value based on longitudinal data and regarding study designs applying also ‘technique-related’ method types. Based on the findings presented above, more ‘talented’ players can be hypothesized to show better technical skills already at young age. While the study by Schorer et al. [20] is one of the first steps in the longitudinal direction, more research is needed to investigate the role of technical skills and the long-term accuracy of TID decisions.

The aim of the current study was to retrospectively investigate 1) the relationships between young handball field players’ technical throwing skills and (a) their potential nomination to youth national teams and (b) their long-term career attainment 10 years later, as well as 2) the associations between nomination status and career attainment, and the effect of nomination status as an additional predictor of career attainment.

## Methods

### Participants

All players (*n* = 478) were chosen by regional coaches to participate in the 2009 national talent selection camp by the German Handball Federation (Deutscher Handballbund e.V.; DHB). Athletes were excluded from the analysis in this study when they were goalkeepers (*n* = 75; 37 females, 38 males) or due to incomplete data sets (*n* = 16). Accordingly, complete data sets were obtained for 193 female (aged 14.5 ± 0.5 years) and 194 male (aged 15.6 ± 0.4 years) handball field players.

### Data Collection

Data collection for this study consisted of two parts. First, players participating in a talent selection camp in 2009 were assessed regarding their throwing skills. In addition, their nomination status for youth national teams during the camp was noted. Second, in August 2020 these players were followed up to determine every player’s highest league of competition achieved in their careers so far. That is, proxies for both short-term (nomination status at the end of the camp) and long-term (career attainment after 10 years) success were collected. More details regarding these two parts of data collection are provided below.

#### Part 1: Short-Term Perspective

During the first part in 2009, data were collected for the players’ throwing skills and nomination status. Data collection was part of a larger assessment by the organizer of the 5-day talent camp (DHB). The one assessment analyzed within this study is the goal throwing skill. Each player conducted ten set-throws from the 9 m-line alternately to target areas (50 cm by 70 cm) in the upper left and upper right corner of the empty goal. Players were instructed to conduct ten throws while also throwing the handballs as powerfully and as precisely as possible toward the target areas. That is, players were assessed executing a highly complex and demanding test comprising both speed and precision pressure. Ball flight velocity for every throw was measured using a radar gun (SpeedTrac X, Arena S.p.A., Tolentino, Italy) placed in the center of the goal (1.5 m behind the goal line and 1 m above ground level) and the average of all ten throws was calculated. Furthermore, digital video cameras (Sony, Sony Corporation, Tokyo, Japan) were used to record the players’ whole-body movement in the sagittal plane (5 m distance) during every throw, allowing for a subsequent technique rating without interfering with camp activities. That is, we followed a both ‘outcome-related’ (precision, throwing velocity, time on exercise) and ‘technique-related’ (technique rating) method type in combination with an ‘experimental’ method setup [16]. The technique rating was conducted independently by two expert raters based on an observation sheet [21]. This observation sheet covered the set-throw including 16 crucial technical elements of the throwing skill while each was rated as 0 when not fulfilled and 1 when fulfilled. Variable technique rating was calculated as the mean of the two raters’ sums. In addition, time on exercise needed for the ten throws was measured (stopwatch) and the number of successful throws hitting the target areas was noted as a measure of precision.

As the talent selection camp’s overall goal was to identify players for youth national teams, some players were nominated to play in those teams by youth national team coaches at the end of the camp. Players were classified as nominated or non-nominated accordingly.

#### Part 2: Long-Term Perspective

For the purpose of identifying the players’ career attainment, each players’ highest league by August 2020 was used as a proxy for their career attainment 10 years after the camp. Players were classified into three groups: (1) professional (first and second German league representing the professional leagues), (2) semi-professional (third German league) and (3) non-professional (fourth German league and lower). Here, being on a team’s roster in the respective league was considered. Players whose highest league could not be identified based on internet research going back to the year 2009 were noted (*n* = 84; 53 females, 31 males) and handled as non-professionals, because all players in the first three German leagues should be appearing in official databases and thus were covered.

### Data Analysis

All statistical analyses were conducted for male and female players separately using IBM SPSS Statistics 27 (IBM Corp., Armonk, New York, USA). Level of significance was set at *α* = 0.05.

Relationships between variables technique rating, precision, throwing velocity and time on exercise were analyzed by Spearman correlation coefficients to check for multi-collinearity.

Binomial logistic regression analyses were conducted to assess the technical variables’ ability to predict the nomination status after camp coded as nominated and non-nominated. Furthermore, multinomial logistic regression analyses were conducted to assess career attainment 10 years after camp coded as professional, semi-professional and non-professional. Here, two different analyses were conducted: one including only the four technical variables and one also including the nomination status as an additional predictor. Effect sizes for all logistic regression analyses (explained variance, *R*^*2*^) were interpreted using Cohen’s [22] conventions (*R*^*2*^ = 0.01–0.089 for small, *R*^*2*^ = 0.09–0.25 for medium, *R*^*2*^ > 0.25 for large effect sizes) as an orientation.

Finally, the association between nomination status and career attainment was tested using 2 × 2 chi-square tests. Again, effect sizes (*w*) were interpreted using Cohen’s [22] conventions (*w* = 0.1–0.29 for small, *w* = 0.3–0.49 for medium, *w* > 0.5 for large) as an orientation.

## Results

First, results of the binomial regression analysis for nomination status are analyzed. Second, findings from the multinomial regression analysis for career attainment are presented. Third, the association between nomination status and career attainment is tested. Finally, the effect of nomination status as an additional predictor of career attainment is tested.

Testing for statistical relationships between all variables found no or only small relationships (*r* ≤ 0.3), suggesting no multi-collinearity exists in the data.

### Descriptives

Table 1 shows the descriptive statistics differentiated by both nomination status at the end of the camp and career attainment for both female and male players.

**Table 1:** Descriptive statistics by nomination status and career attainment for female (top) and male players (bottom)

	Nominated players, *n* = 31	Non-nominated players, *n* = 162	Professional, *n* = 44	Semi-professional, *n* = 23	Non-professional, *n* = 126
**Female**					
Technique rating (Score; max. 16)	11.81 ± 1.55	11.87 ± 1.42	12.05 ± 1.34	12.20 ± 1.35	11.74 ± 1.48
Precision (Score; max. 10)	4.06 ± 1.91	3.75 ± 1.57	4.00 ± 1.70	3.43 ± 1.78	3.79 ± 1.57
Throwing velocity (km/h)	56.84 ± 5.38	53.72 ± 5.33	56.41 ± 5.45	54.67 ± 4.62	53.37 ± 5.40
Time on exercise (s)	27.78 ± 2.63	27.91 ± 3.17	27.34 ± 2.34	27.83 ± 3.32	28.10 ± 3.26

	Nominated players, *n* = 43	Non-nominated players, *n* = 151	Professional, *n* = 52	Semi-professional, *n* = 42	Non-professional, *n* = 100
**Male**					
Technique rating (Score; max. 16)	12.50 ± 1.03	12.39 ± 1.38	12.66 ± 1.38	12.36 ± 1.14	12.31 ± 1.34
Precision (Score; max. 10)	4.12 ± 1.73	3.59 ± 1.80	3.69 ± 1.68	4.24 ± 2.03	3.49 ± 1.73
Throwing velocity (km/h)	65.68 ± 6.64	64.19 ± 6.86	64.13 ± 7.64	66.39 ± 6.69	63.95 ± 6.33
Time on exercise (s)	25.83 ± 2.80	27.41 ± 2.70	27.04 ± 2.52	27.20 ± 3.28	27.01 ± 2.74

Data expressed as mean ± standard deviation

### Retrospective Prediction of Nomination Status

Binomial logistic regression analyses were conducted to retrospectively predict nomination status based on the technical skill variables technique rating, precision, throwing velocity and time on exercise. Table 2 presents the results of the logistic regression model of nomination status for both female and male players.

**Table 2:** Regression analysis of nomination status including technical skill variables

Predictor	Female (*n* = 193)						Male (*n* = 194)					
	B	*SE* B	Wald’s *χ*^*2*^	*df*	*p*	*e*^*B*^ (odds ratio)	B	*SE* B	Wald’s *χ*^*2*^	*df*	*p*	*e*^*B*^ (odds ratio)
Technique rating	-.16	.15	1.14	1	.290	.85	.04	.15	.05	1	.820	1.04
Precision	.09	.12	.48	1	.490	1.09	.10	.10	.93	1	.336	1.10
Throwing velocity	.12	.04	8.98	1	.003	1.13	.06	.03	3.89	1	.049	1.06
Time on exercise	-.01	.07	.01	1	.946	1.00	-.26	.08	11.33	1	.001	.77

The overall model for female players was statistically significant, *χ*^*2*^(4) = 10.64, *p* = 0.03, and explained 9.2% (Nagelkerke *R*^*2*^) of the variance in nomination status while correctly classifying 83.9% of cases. Only variable throwing velocity showed a statistically significant effect (*p* < 0.01, *e*^*B*^ = 1.13).

The overall model for male players was statistically significant, *χ*^*2*^(4) = 17.31, *p* < 0.01, and explained 13.1% (Nagelkerke *R*^*2*^) of the variance in nomination status while correctly classifying 77.8% of cases. Variables throwing velocity and time on exercise showed statistically significant effects while the other variables did not (*p* = 0.049, *e*^*B*^ = 1.06 and *p* < 0.01, *e*^*B*^ = 0.77, respectively).

### Retrospective Prediction of Career Attainment

Table 3 presents the results of the multinomial logistic regression models of career attainment for both female and male players.

**Table 3:** Regression analysis of career attainment including technical skill variables (top), including also nomination status (bottom)

	Predictor	Female (*n* = 193)						Male (*n* = 194)					
		B	*SE* B	Wald’s *χ*^*2*^	*df*	*p*	*e*^*B*^ (odds ratio)	B	*SE* B	Wald’s *χ*^*2*^	*df*	*p*	*e*^*B*^ (odds ratio)
Professional vs. non-professional players	Technique rating	.08	.14	.38	1	.536	1.09	.20	.14	2.16	1	.141	1.23
	Precision	.05	.11	.23	1	.632	1.06	.05	.10	.19	1	.660	1.05
	Throwing velocity	.10	.04	8.21	1	.004	1.11	-.00	.03	.01	1	.915	1.00
	Time on exercise	-.10	.06	2.50	1	.114	.91	.00	.07	.00	1	.994	1.00
Semi-professional vs. non-professional players	Technique rating	.21	.18	1.43	1	.231	1.23	-.10	.15	.43	1	.514	.91
	Precision	- .16	.15	1.11	1	.292	.86	.24	.11	5.10	1	.024	1.28
	Throwing velocity	.03	.05	.45	1	.503	1.03	.05	.03	3.40	1	.065	1.06
	Time on exercise	-.05	.08	.50	1	.480	.95	.02	.07	.08	1	.784	1.02
Professional vs. non-professional players	Nomination status	1.76	.45	15.00	1	< .001	5.79	1.38	.44	10.04	1	.002	3.97
	Technique rating	.16	.15	1.10	1	.294	1.19	.22	.14	2.24	1	.135	1.24
	Precision	.04	.12	.12	1	.730	1.04	.02	.11	.03	1	.874	1.02
	Throwing velocity	.08	.04	3.92	1	.048	1.08	-.02	.03	.36	1	.548	.98
	Time on exercise	-.12	.07	2.89	1	.089	.89	.06	.07	.72	1	.396	1.06
Semi-professional vs. non-professional players	Nomination status	-.90	1.08	.69	1	.407	.41	.55	.49	1.28	1	.258	1.74
	Technique rating	.21	.18	1.42	1	.233	1.24	-.10	.15	.43	1	.513	.91
	Precision	-.17	.15	1.23	1	.267	.85	.23	.11	4.74	1	.030	1.26
	Throwing velocity	.04	.05	.63	1	.428	1.04	.05	.03	2.82	1	.093	1.05
	Time on exercise	-.05	.08	.44	1	.505	.95	.04	.07	.29	1	.589	1.04

Multinomial logistic regression analyses were conducted to retrospectively predict career attainment based on the technical skill variables technique rating, precision, throwing velocity and time on exercise (see Table 3, top).

For female players, the overall multinomial logistic regression model was statistically significant, *χ*^*2*^(8) = 16.24, *p* = 0.04, and explained 9.8% (Nagelkerke *R*^*2*^) of the variance in career attainment while correctly classifying 65.3% of cases. Only variable throwing velocity showed a statistically significant effect for the comparison of professional and non-professional players (*p* < 0.01, *e*^*B*^ = 0.90).

The overall logistic regression model for male players was not statistically significant, *χ*^*2*^(8) = 12.46, *p* = 0.13, and explained 7.1% (Nagelkerke *R*^*2*^) of the variance in career attainment while correctly classifying 54.1% of cases. Also, none of the four variables showed a statistically significant effect.

### Association Between Nomination Status and Career Attainment

**Fig. 1:**
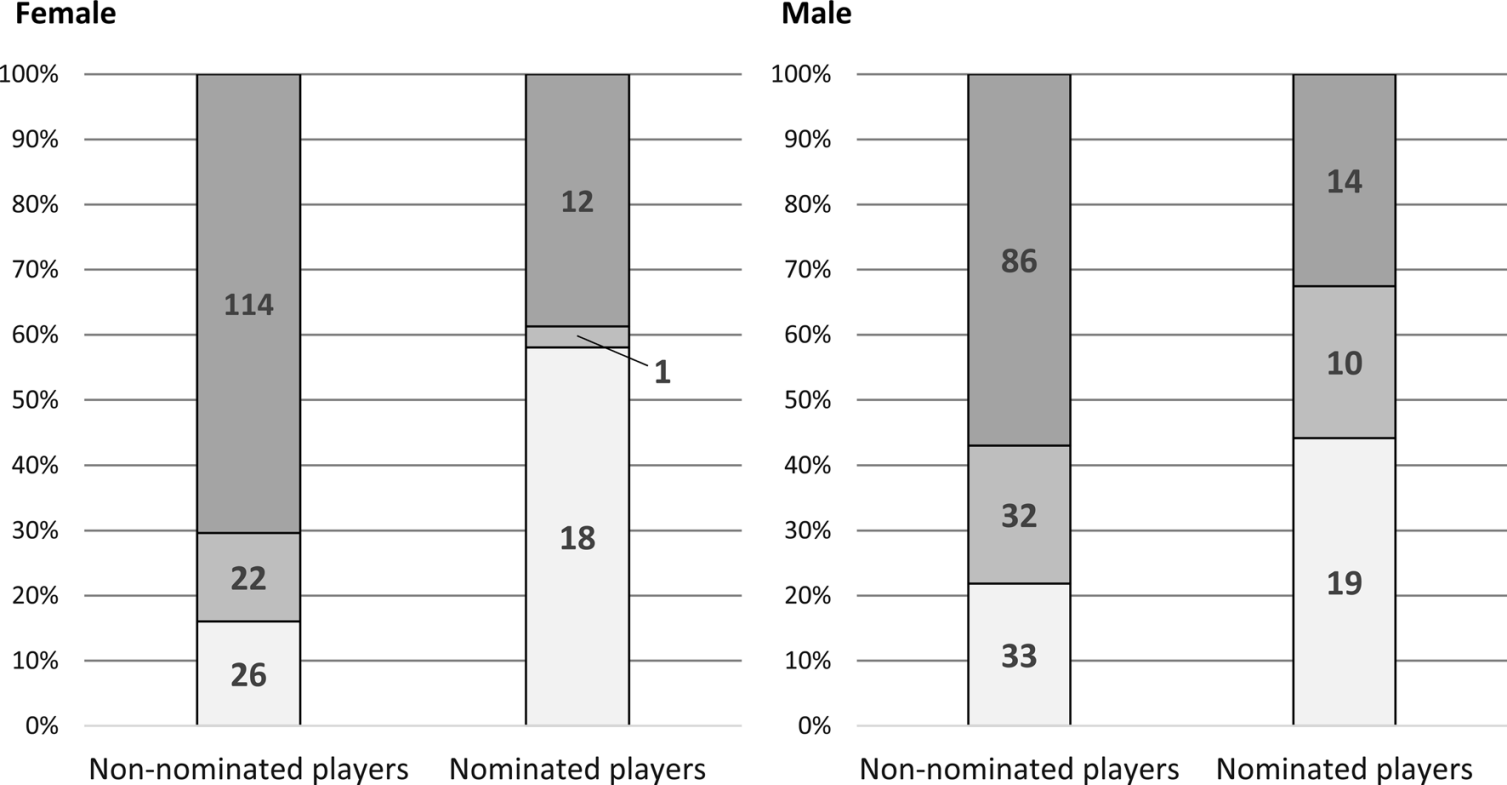
Numbers of female and male players ending up in professional (bottom part in light gray), semi-professional (center part in medium gray) and non-professional handball (upper part in dark gray)

Figure 1 shows the frequency distribution for both female and male players by nomination status at the end of the camp and career attainment.

For female players, a 2 × 2 chi-square test showed a statistically significant association with a medium effect size between nomination status directly at the end of the camp and career attainment 10 years after camp, χ^2^(*df* = 2, *n* = 193) = 26.48, *p* < 0.01, *w* = 0.37. Accordingly, based on the descriptives an Odds ratio of 7.24 was calculated when relating nominated and non-nominated female players making it to the professional level.

Similarly in male players, a 2 × 2 chi-square test showed a statistically significant association with a small effect size, *χ*^*2*^(*df* = 2, *n* = 194) = 10.16, *p* = 0.01, *w* = 0.23. Here, an Odds ratio of 2.83 was calculated.

### Retrospective Prediction of Career Attainment Including Nomination Status

As results showed statistically significant associations between nomination status and career attainment and as the potential nomination happened 10 years before the career attainment assessment, additional multinomial logistic regression analyses were conducted to retrospectively predict career attainment based on not only the four technical skill variables but also nomination status at the end of the camp (see Table 3, bottom).

For female players, the overall multinomial logistic regression model was statistically significant, *χ*^*2*^(10) = 35.27, *p* < 0.01, and explained 20.3% (Nagelkerke *R*^*2*^) of the variance in career attainment. While variable nomination status showed statistically significant effects for the comparison of professional players with both semi-professional and non-professional players (*p* = 0.01, *e*^*B*^ = 0.07 and *p* < 0.01, *e*^*B*^ = 0.17, respectively), variable throwing velocity showed a statistically significant effect for the comparison of professional and non-professional players (*p* = 0.048, *e*^*B*^ = 0.93).

The overall multinomial logistic regression model for male players was statistically significant, *χ*^*2*^(8) = 22.83, *p* = 0.01, and explained 12.7% (Nagelkerke *R*^*2*^) of the variance in career attainment. Here, variable nomination status showed a statistically significant effect when comparing professional and non-professional players (*p* < 0.01, *e*^*B*^ = 0.25).

## Discussion

This study retrospectively investigated (1) the longitudinal relationships between young handball field players’ technical throwing skills and (a) their potential nomination to youth national teams and (b) their long-term career attainment 10 years later, as well as (2) the associations between nomination status and career attainment, and the effect of nomination status as an additional predictor of career attainment were investigated retrospectively.

### Retrospective Prediction of Nomination Status and Career Attainment

In summary of the binomial regression analysis, the statistically significant overall regression model for female players explained 9.2% of the variance in nomination status with a statistically significant effect for variable throwing velocity. On the other hand, the statistically significant overall regression model for male players explained 13.1% of the variance in nomination status with a statistically significant effect for variables throwing velocity and time on exercise. That is, a higher throwing velocity and a lower time on exercise were associated with being nominated to youth national teams.

In summary of the multinomial regression analysis, the statistically significant overall regression model for female players explained 9.8% of the variance in career attainment with a statistically significant effect for variable throwing velocity when comparing professional and non-professional players. In male players, the overall regression model and the variables separately showed no statistically significant effects explaining 7.1% of the variance in career attainment. That is, a higher throwing velocity was associated with becoming a professional female handball player.

Overall, technical skills appear to be on a rather homogenous level for most players in this selected age group. However, variable throwing velocity in both male and especially female players as was previously reported for female players [19] and variable time on exercise in male players showed predictive value. Here, variable time on exercise must be investigated further to check for its validity and practical relevance. Furthermore, higher throwing velocities leave the goalkeeper less time to react to and defend the handball, thus increasing the chance of scoring a goal. Additionally, variable throwing velocity potentially relates to overall power and thus includes other aspects of performance. Here, it is also important to note the effect of potential differences in maturation and growth influencing variable throwing velocity. In general, the high overlaps for technical throwing skills between nominated and non-nominated as well as later professional, semi-professional and non-professional players emphasize the high homogeneity in the already pre-selected sample as players were chosen by regional coaches to participate in the camp. On the one hand, this can be interpreted as high-level technical throwing skills representing an important factor already at regional levels and thus serve rather as a prerequisite than a bonus or key characteristic on national level. Here, probably the players’ age in this study (female age group 14–15, male age group 15–16 years old) also plays an important role as there are big developmental changes in this age group and differences between females and males [e.g., 23]. Especially for the male players in this study, it can be assumed that technical skills play a bigger role at the younger age of 10–14 before movement speed/explosiveness and tactical skills become more important afterward [24]. Thus, technical skills may predict or differentiate better in younger age groups, e.g., in the phase ‘train to learn’ of the long-term athlete development model [25]. Accordingly, the long-term development of handball players should emphasize the improvement of technical (throwing) skills early on by combining it with other performance aspects (e.g., tactical skills). In the beginning, coaches should aim for high amounts of general motor abilities before transferring to more and more handball-specific as well as position-specific skills and drills with older age groups. That is, technical (throwing) skills are an important part within a multidimensional profile and skill set that must be developed in combination with other aspects following the overall aim of ‘playing ability.’ However, it is important to note that while this approach is common practice in many TID systems, its empirical evidence to date is limited.

Furthermore, the assessment of technical skills should include, for example, more than just one throwing technique from one position. Other throwing techniques (e.g., jump shot) and other technical skills (e.g., dribbling, cutting) need to be investigated as potentially not the highly basic skill set-throw but rather throwing variations may differ between players. In addition, not only offensive but also defensive actions as well as different playing positions need to be considered when aiming to improve TID activities as both sides of the game are important [19] and different players have different tasks and rolls demanding different sets of skills and characteristics.

Finally, our results show that technical skills can explain 7–13% of variance in nomination status and career attainment with higher values for the former. That is, about 90% of variance remain unexplained. However, explaining 7–13% of variance with only one performance aspect appears to be a high value, especially for the career attainment with a long study period of 10 years. As Johnston et al. [4] have shown, longitudinal studies (in handball) aiming to explain variance of long-term success are rare. While rather isolated technical skills are a crucial part in the early phases of long-term athlete development programs, they are combined increasingly with tactical skills to train the technical–tactical application of techniques within game contexts. That is, the 87–93% of unexplained variance are probably accounted for by other factors making the multidimensional profile of performance and athlete development in handball. These include performance-related factors such as tactical and psychological skills or mental toughness that are highly important for a long and successful career. This importance of overall athlete development within a TID system is also represented by including nomination status as an additional predictor and finding an increase in explained variance (5.6% for males and 10.6% for females). Also, another factor is injuries that can have negative impacts on a player’s (long term) development and lead to unexpected attainments [26].

### Association Between Nomination Status and Career Attainment

Both chi-square tests showed a statistically significant association between nomination status at the end of the camp and the career attainment 10 years after camp with a medium effect size for female and a small effect size for male players. That is, the nomination status at the end of the camp is associated with the players’ career attainment afterward. This might indicate that coaches are to some degree able to recognize ‘talented’ and in the future successful players as was shown before [e.g., 11]. Vice versa, it also might indicate that the nomination to national teams and with that entering the talent development system of the DHB could increase the chance of becoming a professional player. Here, the multinomial regression analyses for career attainment including nomination status as an additional predictor showing statistically significant effects in combination with highly increased explained variances (9.8% to 20.3% for female, 7.1% to 12.7% for male) probably is an indication that advantages of nomination to the youth national team, e.g., greater quality and quantity of practice, better social networks and surroundings, influence future performance in both female and male handball. As the Odds ratio of the chi-square test show, the chance of making it to the professional level when being nominated 10 years earlier is increased by a factor of 7.24 and 2.83 in female and male players, respectively. Of those 74 players (31 females, 43 males) identified as ‘talented’ and thus nominated by coaches at the camp in 2009, 37 players (18 females, 19 males) ended up playing professional handball while 11 players (1 female, 10 males) played only semi-professionally and 26 players (12 females, 14 males) only non-professionally (see Fig. 1). That is, results show that coaches overall were able to identify (or quasi predict) 38.1% of all later professional handball players (37 of 96) as ‘talented.’ Here, their TID decisions at the camp in 2009 were more ‘successful’ (sensitivity) in females than in males as 58.1% (18 of 31) and 44.2% (19 of 43) of nominated players, respectively, made it to the highest level in their careers. Accordingly, half of the players (37 of 74) identified as ‘talented’ played only semi- or non-professional handball in their careers. The fact that the effect size and the Odds ratios in the chi-square test, the additional explained variance by nomination status in the regression analyses, and the descriptive numbers are higher for females than for males may be explained by the national TID system playing a bigger and more crucial role in female than in male handball. In the latter, there is a more complete and comprehensive talent system at the club level allowing more players to become professional even without the national TID system whereas the national TID system acts as a ‘gatekeeper’ to better development possibilities even more in female handball [27]. Here, further research is needed to investigate the gender data gap in talent research, e.g., regarding differences between female and male TID systems and players’ career chances [28].

### Limitations

Results on inter-rater reliability (intraclass correlation coefficient; ICC) within the technique rating process showed poor reliability (*ICC*[1, 3] = 0.02, *p* = 0.42) between the two raters that might have led to variable technique rating showing no statistically significant effects. Despite similar training beforehand coaches may still have slightly different ideas of throwing technique and assessed certain elements differently, especially within a binary scale. For example, the technical element ‘hand behind the ball’ can be rated differently depending on how far the hand has to be behind the handball to rate this as fulfilled/1 instead of not fulfilled/0. Here, a rating with more than two options could allow for a more detailed assessment. Also, the possibility to assess certain weights to specific elements relating to their importance could improve validity. Furthermore, better training including precise prime examples of categories fulfilled/1 and not fulfilled/0 could improve rater reliability. In addition, rating quality could potentially be increased by presenting videos in slow motion (e.g., 1/4 playback speed) to allow raters to percept movements in more detail [29]. Accordingly, further research regarding the optimal design of observation sheets, quality of video clips, presentation modus, preparation of the coaches and their usage is needed. While other studies showed video observations in combination with technical observations sheets are an effective tool for technique assessment, this has to be restricted based on these findings. Potentially, this downside of observations sheet could be counterbalanced by conducting more objective, biomechanical movement analyses as part of the technique evaluation [30, 31]. Furthermore, future research should consider technique assessments during talent selection activities. However, it is important that assessments are not interfering with camp activities and/or other coach tasks during talent selection.

There are some other limitations to the longitudinal design and analysis of this study. First, changes and developments within a sport and its culture, e.g., tactical approaches or rule adaptions, can influence the role of certain skills or characteristics over time, especially when analyzing data longitudinally. Second, data on maturation and growth as well as potential ‘relative age effects’ were not analyzed in this study, but could have affected the technical throwing skill assessments, e.g., differences in throwing velocity. Future studies should include this information in their analysis. Third, potentially there are errors with some of our assessment tools. Especially the variable technique rating and its assessment process based on an observation sheet must be advanced and improved to allow for high-quality data from a ‘technique-related’ method type. In addition, using the players’ highest league as a proxy for career attainment may have reduced the players’ performance to a rather tight variable. Here, future studies should consider other measurements of career attainment, e.g., games played [13], titles won, goals scored or other game statistics. Fourth, as mentioned above, future studies should include technical skills as one dimension in a multidimensional approach. Last, including players with no information on their highest league in the group of non-professional players during the analysis may have introduced some error and future studies could try to incorporate potential reasons for lower career attainments, e.g., injury.

## Conclusion

All in all, this study shows that technical throwing skills are at a very homogeneous high-level throughout participants at the German national talent level (female age group 14–15, male age group 15–16 years old). However, some associations were found comparing players regarding their nomination status and career attainment. Here, variable throwing velocity showed the biggest effects. In addition, this study shows the importance of being nominated to youth national teams (i.e., entering the national TID system) for career attainment. This emphasizes the importance of longitudinal and comprehensive approaches in TID and the need for improvement in ‘technique-related’ assessment tools, e.g., observation sheets or biomechanical movement analysis. Here, combinations of various (subjective and objective) assessment methods appear promising within multidimensional assessment approaches [12]. Furthermore, the importance and weight of specific skills and aspects in certain age groups must be investigated to adapt TID activities to the young players’ development. Finally, more longitudinal assessments should be conducted to get deeper insights into the predictive power of various talent aspects.

## Data Availability

Datasets generated and/or analyzed in the context of the current study cannot be made publicly available for ethical and legal reasons; the public availability would compromise confidentiality and/or participant privacy as the data contain potentially identifying information.

## Abbreviations

DHB: Deutscher Handballbund e.V. (German Handball Federation)
TID: Talent identification and development
